# Effects of Exercise on Bone Marrow Adipose Tissue in Children With Overweight/Obesity: Role of Liver Fat

**DOI:** 10.1210/clinem/dgae547

**Published:** 2024-08-07

**Authors:** Idoia Labayen, Cristina Cadenas-Sánchez, Fernando Idoate, Luis Gracia-Marco, María Medrano, Víctor Manuel Alfaro-Magallanes, Juan M A Alcantara, Beatriz Rodríguez-Vigil, Maddi Osés, Francisco B Ortega, Jonatan R Ruiz, Rafael Cabeza

**Affiliations:** Institute for Sustainability & Food Chain Innovation (IS-FOOD), Department of Health Sciences, Public University of Navarre, 31006 Pamplona, Navarre, Spain; IdiSNA, Navarra Institute for Health Research, 31006 Pamplona, Navarre, Spain; Centro de Investigación Biomédica en Red Fisiopatología de la Obesidad y Nutrición (CIBERobn), Instituto de Salud Carlos III, 28029 Madrid, Spain; Institute for Sustainability & Food Chain Innovation (IS-FOOD), Department of Health Sciences, Public University of Navarre, 31006 Pamplona, Navarre, Spain; IdiSNA, Navarra Institute for Health Research, 31006 Pamplona, Navarre, Spain; Centro de Investigación Biomédica en Red Fisiopatología de la Obesidad y Nutrición (CIBERobn), Instituto de Salud Carlos III, 28029 Madrid, Spain; Department of Physical Education and Sports, Faculty of Sport Sciences, Sport and Health University Research Institute (iMUDS), University of Granada, 18007 Granada, Spain; Radiology Department, Mutua Navarra, Department of Health Sciences, Public University of Navarre, 31012 Pamplona, Navarre, Spain; Centro de Investigación Biomédica en Red Fisiopatología de la Obesidad y Nutrición (CIBERobn), Instituto de Salud Carlos III, 28029 Madrid, Spain; Department of Physical Education and Sports, Faculty of Sport Sciences, Sport and Health University Research Institute (iMUDS), University of Granada, 18007 Granada, Spain; Instituto de Investigación Biosanitaria, ibs.Granada, 18012 Granada, Spain; Institute for Sustainability & Food Chain Innovation (IS-FOOD), Department of Health Sciences, Public University of Navarre, 31006 Pamplona, Navarre, Spain; IdiSNA, Navarra Institute for Health Research, 31006 Pamplona, Navarre, Spain; Centro de Investigación Biomédica en Red Fisiopatología de la Obesidad y Nutrición (CIBERobn), Instituto de Salud Carlos III, 28029 Madrid, Spain; Institute for Sustainability & Food Chain Innovation (IS-FOOD), Department of Health Sciences, Public University of Navarre, 31006 Pamplona, Navarre, Spain; IdiSNA, Navarra Institute for Health Research, 31006 Pamplona, Navarre, Spain; Department of Health and Human Performance, Faculty of Physical Activity and Sport Sciences, LFE Research Group, Universidad Politécnica de Madrid, 28040 Madrid, Spain; Institute for Sustainability & Food Chain Innovation (IS-FOOD), Department of Health Sciences, Public University of Navarre, 31006 Pamplona, Navarre, Spain; IdiSNA, Navarra Institute for Health Research, 31006 Pamplona, Navarre, Spain; Centro de Investigación Biomédica en Red Fisiopatología de la Obesidad y Nutrición (CIBERobn), Instituto de Salud Carlos III, 28029 Madrid, Spain; Department of Magnetic Resonance Imaging, University Hospital of Araba (HUA), Osakidetza Basque Health Service, Osatek, Bioaraba Health Research Institute, 01004 Vitoria-Gasteiz, Alava, Spain; Institute for Sustainability & Food Chain Innovation (IS-FOOD), Department of Health Sciences, Public University of Navarre, 31006 Pamplona, Navarre, Spain; IdiSNA, Navarra Institute for Health Research, 31006 Pamplona, Navarre, Spain; Centro de Investigación Biomédica en Red Fisiopatología de la Obesidad y Nutrición (CIBERobn), Instituto de Salud Carlos III, 28029 Madrid, Spain; Department of Physical Education and Sports, Faculty of Sport Sciences, Sport and Health University Research Institute (iMUDS), University of Granada, 18007 Granada, Spain; Faculty of Sport and Health Sciences, University of Jyväskylä, 40500 Jyväskylä, Finland; Centro de Investigación Biomédica en Red Fisiopatología de la Obesidad y Nutrición (CIBERobn), Instituto de Salud Carlos III, 28029 Madrid, Spain; Department of Physical Education and Sports, Faculty of Sport Sciences, Sport and Health University Research Institute (iMUDS), University of Granada, 18007 Granada, Spain; Instituto de Investigación Biosanitaria, ibs.Granada, 18012 Granada, Spain; Department of Electrical, Electronic and Communications Engineering, Smart Cities Institute, Public University of Navarre, 31006 Pamplona, Spain

**Keywords:** bone health, obesity, fatty liver, MAFLD, MASLD, NAFLD, youth, lifestyle

## Abstract

**Context:**

Exercise reduces adiposity, but its influence on bone marrow fat fraction (BMFF) is unknown; nor is it known whether a reduction in liver fat content mediates this reduction.

**Objectives:**

This work aimed to determine whether incorporating exercise into a lifestyle program reduces the lumbar spine (LS) BMFF and to investigate whether changes in liver fat mediate any such effect.

**Methods:**

Ancillary analysis of a 2-arm, parallel, nonrandomized clinical trial was conducted at primary care centers in Vitoria-Gasteiz, Spain. A total of 116 children with overweight/obesity were assigned to a 22-week family-based lifestyle program (control group [n = 57]) or the same program plus an exercise intervention (exercise group [n = 59]). The compared interventions consisted of a family-based lifestyle program (two 90-minute sessions/month) and the same program plus supervised exercise (three 90-minute sessions/week). The primary outcome examined was the change in LS-BMFF between baseline and 22 weeks, as estimated by magnetic resonance imaging. The effect of changes in hepatic fat on LS-BMFF were also recorded.

**Results:**

Mean weight loss difference between groups was 1.4 ± 0.5 kg in favor of the exercise group. Only the children in the exercise group experienced a reduction in LS-BMFF (effect size [Cohen *d*] −0.42; CI, −0.86 to −0.01). Importantly, 40.9% of the reductions in LS-BMFF were mediated by changes in percentage hepatic fat (indirect effect: β=−0.104; 95% CI, −0.213 to −0.019). The effect of changes in hepatic fat on LS-BMFF was independent of weight loss.

**Conclusion:**

The addition of exercise to a family-based lifestyle program designed to reduce cardiometabolic risk improves bone health by reducing LS-BMFF in children with overweight or obesity. This beneficial effect on bone marrow appears to be mediated by reductions in liver fat.

Bone marrow adipose tissue (BMAT) is localized within the bone cavity as a component of the bone marrow. BMAT undergoes expansion during skeletal growth and development, a phenomenon that accelerates during aging and menopause. In adults, it accounts for approximately 70% of the bone marrow volume, and makes up roughly 10% of total body adipose tissue (1). BMAT is a unique environment where adipocytes and bone cells share the milieu. Clinical studies have indicated that BMAT content is inversely correlated with bone mineral density (BMD) and a higher incidence of vertebral fractures (2); reducing BMAT might therefore help in the prevention and treatment of osteoporosis.

Children with overweight/obesity are at increased risk of fractures (3); however, the mechanism(s) underlying this is not understood. Interestingly, several studies in adults with morbid obesity (4) or type 2 diabetes (5), and others performed in children (6), have reported the bone marrow fat fraction (BMFF) to be inversely associated with BMD. This implies that the accumulation of BMAT may occur at the expense of osteoblasts, potentially impeding bone formation and resulting in reduced BMD in adults, and delayed or smaller BMD gain in children. Recent work by our group substantiates these observations and expands them to encompass children with overweight/obesity (7).

In adults, there is very limited research examining the effect of pharmacological (8), lifestyle-based, or surgery-induced weight loss interventions on BMAT. Overall, the results of dietary- or surgical-based trials show that although weight loss was associated with fat loss, the reduction in BMAT did not always follow changes in body mass or adiposity (9-11). Thus, the influence of diet and exercise on BMAT remains unclear, but multicomponent intervention programs including the promotion of a healthy lifestyle and exercise training are effective in preventing and treating pediatric obesity and its comorbidities (12). However, physical activity and exercise might help protect the bone marrow milieu, suppressing the formation of BMAT and preserving the regenerative osteogenic function of mesenchymal cells mediated by osteoblasts (13). It is therefore reasonable to hypothesize that exercise might reduce BMAT in children with overweight/obesity, but clinical studies that support this idea are lacking.

There is also evidence of a metabolic link between liver fat content and bone health in children and adolescents. Two studies in children report that liver fat content is inversely associated with BMD, independently of adiposity (14, 15). Importantly, in a cross-sectional study, our group concluded that the observed relationship between hepatic fat and BMD was likely mediated via the lumbar spine (LS) BMFF (7), supporting the idea that metabolic crosstalk occurs between hepatic adipose tissue and LS-BMFF. Given these findings, the present work aimed to (i) analyze whether a multicomponent intervention program (designed according to current evidence and guidelines (16)) including a family-based lifestyle and psychoeducation program plus a supervised exercise intervention was more effective at reducing LS-BMFF than the lifestyle program alone, and (ii), to determine whether the effect of the intervention on LS-BMFF is mediated by changes in liver fat content in children with overweight/obesity.

The present work is an ancillary analysis of the EFIGRO project, whose main finding is that the addition of exercise training to a family-based lifestyle intervention program results in a significant liver fat reduction (∼20%) and greater loss of adiposity and visceral adipose tissue (VAT) (12).

## Materials and Methods

### Study Design and Participants

The present study is a secondary analysis conducted within the framework of the EFIGRO project (ClinicalTrials.gov. ID NCT02258126) (17). The EFIGRO project is a 2-arm, parallel-design, nonrandomized controlled trial that was undertaken between 2014 and 2017 in Vitoria-Gasteiz, Spain. The study protocol was approved by the Euskadi Clinical Investigation Ethics Committee (PI2014045) and was performed according to the ethical guidelines of the Declaration of Helsinki (2013 revision). Parents or legal guardians gave their written, informed consent for their children/charges to take part, and all the children gave their assent before enrollment. The trial protocol (17) and main effects of the clinical trial have been published elsewhere (12, 18-20). Briefly, the children and their families were recruited at the Paediatric Endocrinology Unit of the University Hospital of Araba, and from primary care clinics, in Vitoria-Gasteiz. The main entry criterion was showing overweight or obesity, as defined according to the World Obesity Federation (21). In addition, the children had to be between ages 8 and 12 years, and to have at least 1 parent or caregiver willing to participate in the program. Children whose medical condition limited their physical activity or that might affect the results of the study were excluded. In addition, according to the recommendations (22, 23), children with liver disease or and any other problem accompanied by elevated blood transaminase levels, such as viral hepatitis, toxic hepatitis, or autoimmune disease, also were excluded.

**Figure 1.**
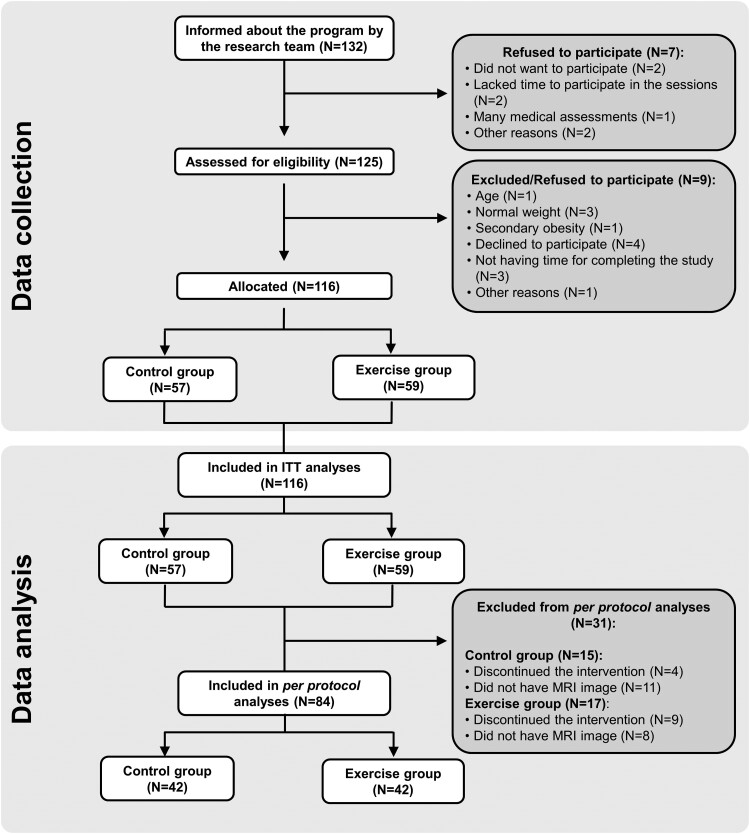
Flowchart of children participating in the study. Control group: children participating in the family-based lifestyle and psychoeducation program; exercise group: children participating in the latter plus exercise training. BMFF, bone marrow fat fraction.

A total of 116 children (aged 10.6 ± 1.1 years, 53.4% girls) were allocated to either a group receiving a family-based lifestyle and psychoeducation program (hereinafter referred as the control group, N = 57) or another group receiving the same program plus an exercise training program (hereinafter referred as the exercise group, N = 59). Figure 1 shows the participation flowchart following Consolidated Standards of Reporting Trials (CONSORT) guidelines (24). Additionally, the trial followed the Transparent Reporting of Evaluations with Nonrandomized Designs (TREND) reporting guidelines (25).

### Family-based Healthy Lifestyle Program

Parents and children from both the control and exercise groups participated in 2 monthly 45-minute sessions over a 22-week program focused on promoting a healthy lifestyle (total 11 sessions) along with a psychoeducation program (45 minutes/session, 11 sessions) designed and conducted by experienced nutritionists and psychologists, respectively. The goals of the program were to improve the lifestyle habits and psychological well-being of the children, to optimize the family environment for making positive changes, and to provide assertive communication skills. Detailed information on the design, aims, and content of the intervention has been published elsewhere (17).

### Exercise Training Program

In addition to the aforementioned portion of the program, the children in the exercise group participated in an exercise program (designed and supervised by exercise specialists) involving 3 weekly sessions (90 minutes/session) over the 22-week intervention period. The program mainly focused on high-intensity, aerobic game-based workouts (>76% heart rate peak), but also included muscle strength and stretching exercises following World Health Organization guidelines (26). Exercise intensity was controlled during the sessions using Polar RS300X heart rate monitors (Polar Electro). The full design of the program is available elsewhere (17). The mean attendance of the exercise program sessions was 72.0 ± 16.1%. The average heart rate per session was 146 ± 16 bpm. High-intensity exercise (ie, ≥ 77% of maximum heart rate) was maintained during 49 ± 23%, and moderate intensity (ie, 64%-76% of maximum heart rate) 32 ± 15% of the time during the workout sessions.

### Measurements

All variables were measured at baseline and after the 22-week intervention by the same researchers. Postintervention evaluations were scheduled within 3 days of the last healthy lifestyle or exercise session.

LS-BMFF, percentage hepatic fat and VAT (cm^2^) were measured using a MAGNETOM Avanto 1.5T system (Siemens Healthcare) equipped with a phased-array surface coil and a spine array coil. Percentage hepatic fat was measured using the Siemens Medical System software v.syngo.MR B17 following a 6-point Dixon technique. Thereafter, children were categorized into 2 groups according to the presence or absence of hepatic steatosis (<5.5 or ≥5.5% hepatic fat, respectively). VAT was calculated using a semi-automatic software for fat segmentation. Two independent radiologists performed a manual delineation of the L1 to L4 vertebra using MANGO software (http://ric.uthscsa.edu/mango/). Once the contours were obtained, the fat fraction image was examined by MATLAB software to obtain the mean and SD values for the fat fraction of each vertebra. Detailed information on the quantification of VAT and LS-BMFF can be found elsewhere (7, 18). The medical imaging group was totally blinded to the intervention group.

Dual energy x-ray absorptiometry was used to measure total body fat mass and lean mass (HOLOGIC QDR 4500W). The fat mass index (FMI) and lean mass index were calculated as fat mass and lean mass divided by height squared (kg/m^2^), respectively.

### Statistical Analyses

Power calculation and sample size estimations were calculated based on the primary outcome of the EFIGRO trial (17). The present work is based on a secondary analysis and, therefore, no specific power calculation was performed. The distribution of the variables was verified using the Shapiro-Wilk test, skewness, and kurtosis values. Variables with nonnormal distributions (ie, percentage hepatic fat and VAT) were log-transformed. Descriptive outcomes were reported as means and SDs.

Within-group differences (pre vs post values) in LS-BMFF were examined using the paired *t* test. The effect of the intervention (post minus pre intervention values) between the control and exercise groups in LS-BMFF was examined by analysis of covariance adjusting for age, sex, and baseline LS-BMFF.

The mediating effect of changes in percentage hepatic fat on changes in LS-BMFF was examined using the PROCESS macro for SPSS with 10 000 bootstrap samples. Differences were deemed statistically significant when the indirect effect significantly differed from zero. As a sensitivity analysis, the mediating effects of changes in weight, VAT, and FMI instead of percentage hepatic fat were also explored.

Differences were also sought in terms of changes in LS-BMFF between percentage hepatic fat responders and nonresponders. Children were considered responders or nonresponders when the change in percentage hepatic fat after the intervention was greater than or equal to 0.2 or less than 0.2 of the effect size (*d*-Cohen), respectively.

All analyses were performed per protocol (ie, children who successfully completed the trial attending at least 50% of the family-based lifestyle and psychoeducation intervention sessions) and following intention-to-treat principles (including all children that started the trial, whether or not they completed 50% of the lifestyle and psychoeducation intervention sessions). For the intention-to-treat analyses, missing values at follow-up were computed by multiple imputation. Since sex had no effect on changes in LS-BMFF or percentage hepatic fat, the results for girls and boys were analyzed together. All analyses were undertaken using the IBM Statistical Package for Social Sciences (SPSS v. 24.0 for Windows) (IBM Corp). Significance (2-tailed) was set at *P* less than .05.

## Results

Table 1 presents the characteristics of the participating children. The effects of the intervention on body weight and composition are displayed in Table 2.

**Table 1.** Baseline characteristics of children participating in the study

	Whole sample (N = 116)		Control group (N = 57)		Exercise group (N = 59)	
	Mean	SD	Mean	SD	Mean	SD
Age, y	10.5	1.1	10.6	1.1	10.5	1.1
Girls (n, %)	64	53.4	30	52.6	32	54.2
Body mass index	25.4	3.2	25.2	2.8	25.7	3.7
Fat mass index	10.1	2.3	9.8	2.1	10.5	2.6
Lean mass index	14.5	1.3	14.7	1.2	14.4	1.3
Lumbar spine BMFF, %	44.9	9.3	44.9	10.3	45.0	8.3
Abdominal VAT, cm^2^	46.3	20.7	44.2	20.8	46.9	20.1
Hepatic fat, %	5.7	4.1	5.3	2.9	6.0	4.8

Abbreviations: BMFF, bone marrow fat fraction, VAT, visceral adipose tissue.

**Table 2.** Changes in body weight and composition in the control and exercise groups at the end of the intervention

	Control group		Exercise group	
	Mean	SD	Mean	SD
Δ Body weight, kg	0.5	2.4	−0.9	2.6
Δ Body mass index	−0.67	1.00	−1.30	1.13
Δ Fat mass index	−0.84	0.91	−1.11	0.81
Δ Lean mass index	−0.11	0.53	−0.05	0.48
Δ Abdominal VAT, cm^2^	−3.7	8.4	−8.0	7.7
Δ Hepatic fat (%)	0.04	1.87	−1.15	2.43

Abbreviation: VAT, visceral adipose tissue.

ΔPostintervention minus preintervention values. These data were presented in our previous article reporting the main intervention effects (12).

### Effects of Intervention on Bone Marrow Adipose Tissue

**Figure 2.**
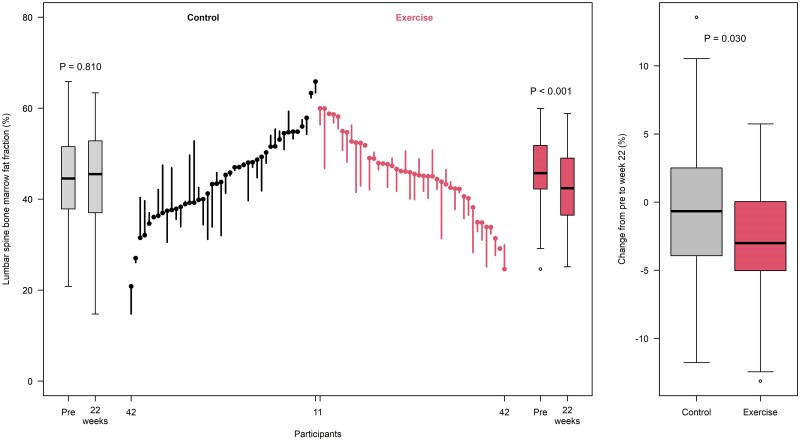
Lumbar spine bone marrow fat fraction before (Pre) and after (22 weeks) participation in the family-based lifestyle and psychoeducation program (control, black), or the same plus exercise training (exercise group, red); results are for every participating child. The ends of the boxes in the box plots are located at the first and third quartiles; the black line in the middle denotes the median. Whiskers extend to the upper and lower adjacent values, the location of the furthest point within a distance of 1.5 interquartile ranges from the first and third quartiles. The parallel line plot shows one vertical line for each participant, extending from their baseline to their 22-week value. Descending lines indicate a reduction in the lumbar spine bone marrow fat fraction, whereas ascending lines indicate an increase. Pretest values are placed in ascending order for the control group and descending order for the exercise group. Intragroup (pre vs post) differences were calculated using the paired *t* test. Intergroup (control vs exercise) differences (post minus pre) were calculated by one-way analysis of covariance adjusted for baseline value, age, and sex. The data used for the figure can be found in eTable 1.

Figure 2 shows the changes in LS-BMFF from baseline to 22 weeks for every child in the control and exercise groups, as well as the difference between these groups. At the end of the intervention, LS-BMFF was reduced only in the exercise group (*P* < .001); the difference between the groups in terms of this variable (determined per protocol) was also significant (−1.9% [95% CI, −3.2 to −0.05]; *P* = .030; Cohen *d*: −0.42). The intention-to-treat analysis returned similar, although slightly attenuated, results (−1.1% [95% CI, −2.1 to −0.01]; *P* = .051; Cohen *d*: 0.37; Supplementary Table S1). eTable 1 shows the effect of the intervention on the members of the control and the exercise groups as determined per protocol and intention to treat (27).

### Liver Fat Content Mediates Reduction in Bone Marrow Adipose Tissue

**Figure 3.**
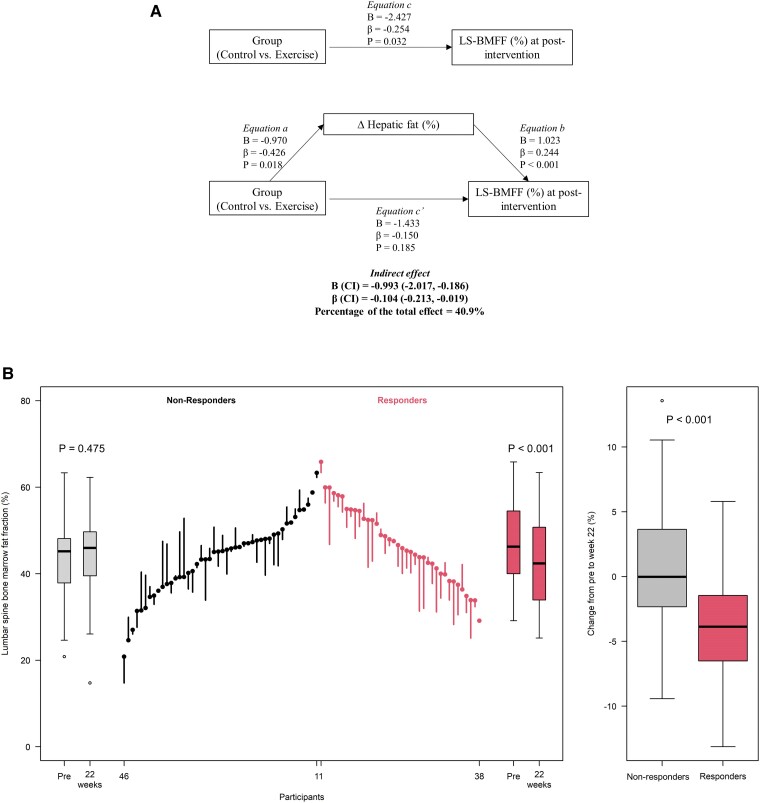
A, Mediation analysis. Effects of the intervention on changes in bone marrow fat fraction (BMFF) through changes in percentage hepatic fat, adjusting for confounders (baseline values, age, and sex). B, Lumbar spine BMFF before (Pre) and after (22 weeks) for every child with overweight/obesity who experienced a reduction (*d*-Cohen ≥ 0.2) (responders), or not (nonresponders, *d*-Cohen < 0.2), in percentage hepatic fat by the end of the intervention. The ends of the boxes in the box plots are located at the first and third quartiles; the black line in the middle denotes the median. Whiskers extend to the upper and lower adjacent values, the location of the furthest point within a distance of 1.5 interquartile ranges from the first and third quartiles. The parallel line plot contains one vertical line for each participant, extending from their baseline to their 22-week value. Descending lines indicate a reduction in the lumbar spine bone marrow fat fraction whereas ascending lines indicate an increase. Pretest values are placed in ascending order for non-responders and descending order for responders. Intragroup (pre vs post) differences were calculated using the paired *t* test. Intergroup (control vs exercise) differences were calculated by one-way analysis of covariance adjusted for baseline value, age, and sex. The data used for Fig. 3B can be found in eTableS2.

Figure 3A shows the mediation model linking changes in percentage hepatic fat to changes in LS-BMFF. A total of 40.9% of the intervention-induced reductions in LS-BMFF were mediated by the reduction in percentage hepatic fat (indirect effect: β=−0.104; 95% CI, −0.213 to −0.019). In addition, only those children who experienced a reduction in percentage hepatic fat at the end of the intervention (ie, responders) showed a statistically significant reduction in LS-BMFF (*P* < .001; Fig. 3B). At the end of the intervention, the difference in the reduction in LS-BMFF between percentage hepatic fat responders and nonresponders (−3.9% ± 0.7% compared to 0.4% ± 0.7%) was statistically significant (age, sex, and baseline BMFF adjusted *P* < .001; Fig. 3B). eTable 2 shows the effect of the intervention in nonresponders and responders as determined per protocol and intention to treat (27). When substituting changes in percentage liver fat for changes in body weight (β=−0.502; 95% CI, −1.633 to 0.149), VAT (β=−0.104; 95% CI, −0.347 to 0.036; *P* = .152) or FMI (β=−0.031; 95% CI, −0.182 to 0.075; *P* = .212) in the per protocol mediation analysis, no mediation was detected. Furthermore, adjusting for changes in body weight slightly increased the mediating effect of changes in liver fat on LS-BMFF (β=−0.833; 95% CI, −2.015 to−0.197). Thus, we observed that changes in liver fat accounted for 43.2% of the reduction in LS-BMFF, independent of body weight loss.

The intention-to-treat analyses yielded similar results. Specifically, changes in percentage hepatic fat accounted for 38.3% of the reduction in LS-BMFF (indirect effect: β = −0.068; 95% CI, −0.141 to −0.012; eFig. 1) (27). Further, the percentage of hepatic fat responders also showed a significant reduction in LS-BMFF (*P* < .001; eTable 2) (27), which was notably greater than the reduction observed in nonresponders (*P* < .001; see eTable 2) (27).

## Discussion

The present study shows that the addition of exercise to a 22-week lifestyle education program significantly reduces LS-BMFF in children with overweight/obesity and, importantly, that this reduction greatly depends on an improvement in liver fat content independent of body weight loss. As far as we are aware, this is the first study to examine the effects of a lifestyle intervention program designed according to the international guidelines for childhood obesity management on BMAT in children with overweight/obesity. The results add to the evidence provided by studies performed in adults (28, 29) that suggest the existence of a metabolic relationship between liver fat content and bone health, and that reducing hepatic fat may provide a means of preventing and treating osteoporosis (30).

Family-based lifestyle programs are the cornerstone of childhood treatment (31). These interventions can reduce overall adiposity and cardiovascular risk factors such as insulin resistance (18), but the reduction of certain ectopic body fat deposits, such as in the liver, requires physical exercise (12). In the present work, the lifestyle education program alone did not reduce LS-BMFF; a reduction was observed only in those who also engaged in the supervised exercise program. These results are consistent with those of a prior, pilot study involving preschool children (32), in which a 10-week intervention of school-based physical activity led to reductions in femoral BMAT (which increased in an age- and sex-matched control group). The present findings take on special importance in the context of the inverse association previously recorded between LS-BMFF and BMD (7) in a population at high risk of fractures and osteoporosis.

Hepatic fat content thus appears to have a considerable effect on bone health, likely via some still unknown metabolic interplay. The reduction in LS-BMFF seen in the children who followed the exercise intervention program depended on the improvement in liver fat content. Notably, mediation analyses indicated that the reduction in percentage hepatic fat accounted for 40% of the reduction in LS-BMFF. Moreover, only children who experienced a meaningful improvement in percentage hepatic fat (ie, responders) showed a reduction in LS-BMFF. Taken together, these findings may explain previously observed inverse associations between liver fat content and BMD (14). Certainly, they highlight the importance of reducing hepatic fat to improve liver and cardiometabolic health, and to improve bone health, in the first decade of life.

Exercise-induced reduction in liver fat may decrease LS-BMFF, potentially due to elevated insulin-like growth factor-1 (IGF-1). IGF-1, a liver-derived hormone, reduces osteoblast apoptosis, promotes osteoblastogenesis, and regulates BMAT (33, 34). Higher IGF-1 levels are linked to lower BMAT, as seen in bariatric surgery patients and premenopausal women (35). Mouse studies show reduced IGF-1 increases bone marrow adipogenic potential (36). Further research is needed to confirm if increased IGF-1 explains the observed effects.

Interestingly, in our study, weight loss did not mediate the reduction of LS-BMFF. Moreover, the effect of reducing hepatic fat was independent of body weight loss. Studies on lifestyle intervention–induced weight loss and its effects on BMFF are limited and show contradictory results. Some studies found that weight loss does not always reduce BMAT. For example, Cordes et al (37) saw no significant change in LS-BMFF in obese women after a dietary intervention that resulted in a mean weight loss of approximately 7 kg, while Vogt et al (9) reported reductions in body mass index, liver fat (from 14.2% to 4.1%), and BMFF (∼5% decrease) in patients with obesity and type 2 diabetes after formula diet intervention. Unfortunately, the correlations among these changes were not analyzed. Ofir et al (10) examined the effects of a hypocaloric diet intervention, both with and without physical activity, on LS-BMFF. They observed reductions in LS-BMFF that were not correlated with changes in other fat depots. However, the differences between the two groups were not reported.

One of the strengths of this study is the use of magnetic resonance imaging for measuring LS-BMFF and liver fat content. Moreover, the sample size was larger, and the duration of the intervention program longer than in a previous study in children (31). The most important limitation of the present work is that the randomization process could not be followed as planned. The EFIGRO project was initially conceived as a randomized clinical trial, but complete randomization when assigning participants to the control or the exercise groups was not feasible: Some children (and families) were unable to attend the exercise sessions due to time constraints. The decision was made not to exclude them (N = 11) but to assign them to the control group. This course of action was chosen since the children/families had been encouraged to join the study by their pediatricians, who highlighted the potential health benefits associated with the program. It is worth noting that the participants in both groups were comparable in terms of body mass index, age, and percentage hepatic fat, etc, at baseline, and attempts were made to adjust for any differences.

## Conclusion

The present findings suggest that the addition of exercise to a lifestyle intervention program designed to reduce cardiometabolic risk improves bone health by reducing LS-BMFF in children with overweight or obesity. Moreover, the effect of exercise on LS-BMFF was mediated by reductions in liver fat, independent of changes in body mass loss or other specific fat depots. Further studies are needed to corroborate our results.

## Data Availability

Due to privacy concerns, the data sets used in this study are not publicly available. However, specific datasets can be made available on request for academic use only for 36 months from the date of publication, following deidentification. Proposals should be directed to idoia.labayen@unavarra.es. On proposal acceptance, requestors will be granted access to the data after signing a data access agreement.

## Clinical Trial Information

ClinicalTrials.gov ID number NCT02258126 (registered April 1, 2019).

## Abbreviations

BMAT: bone marrow adipose tissue
BMD: bone mineral density
BMFF: bone marrow fat fraction
FMI: fat mass index
IGF-1: insulin-like growth factor-1
LS: lumbar spine
VAT: visceral adipose tissue
